# Supplementary figures and images for: Streptococcus agalactiae Induces Placental Macrophages To Release Extracellular Traps Loaded with Tissue Remodeling Enzymes via an Oxidative Burst-Dependent Mechanism
Source: mBio. 2018 Nov 20;9(6):e02084-18. doi: 10.1128/mBio.02084-18 (PMC6247082; doi:10.1128/mBio.02084-18)

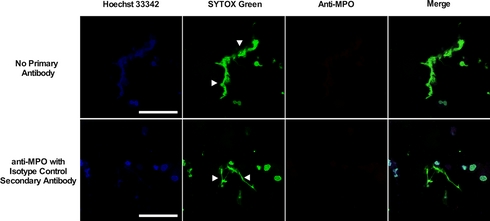

Supplement: FIG S1 [file mbo006184186sf1.jpg]

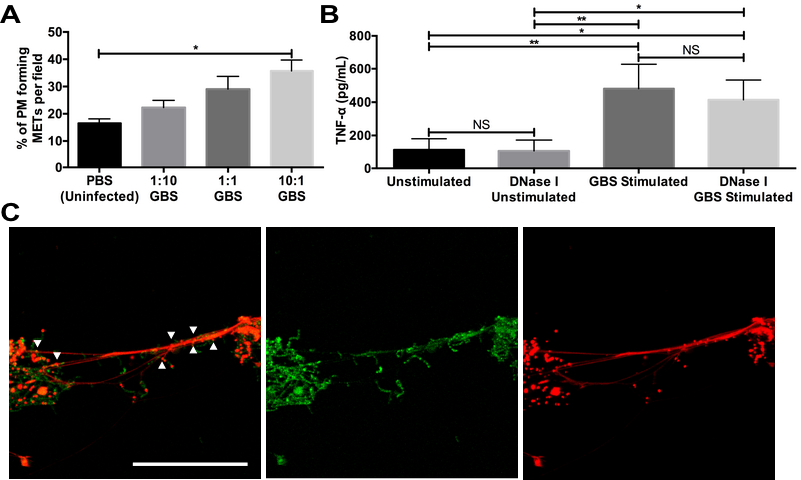

Supplement: FIG S2 [file mbo006184186sf2.tif]

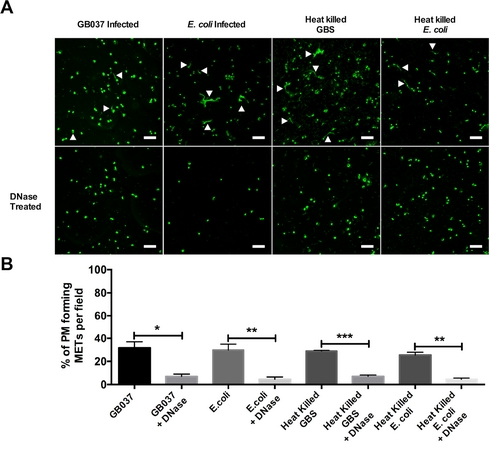

Supplement: FIG S3 [file mbo006184186sf3.jpg]

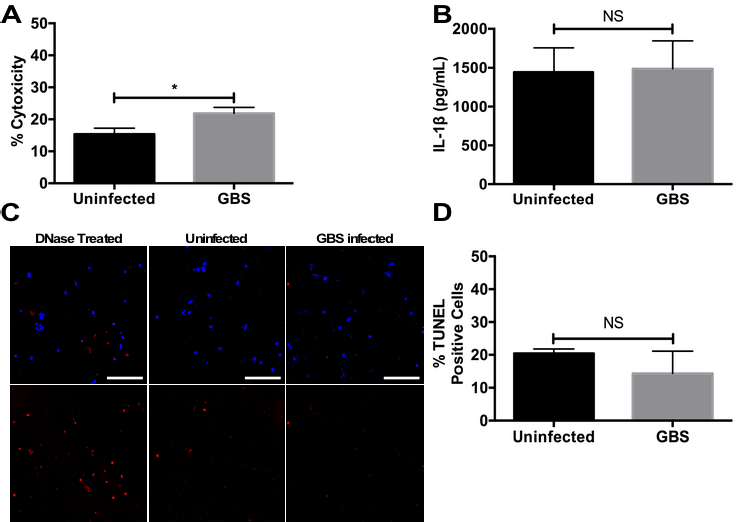

Supplement: FIG S4 [file mbo006184186sf4.tif]

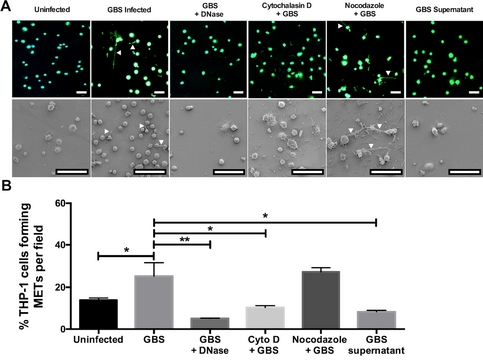

Supplement: FIG S5 [file mbo006184186sf5.jpg]

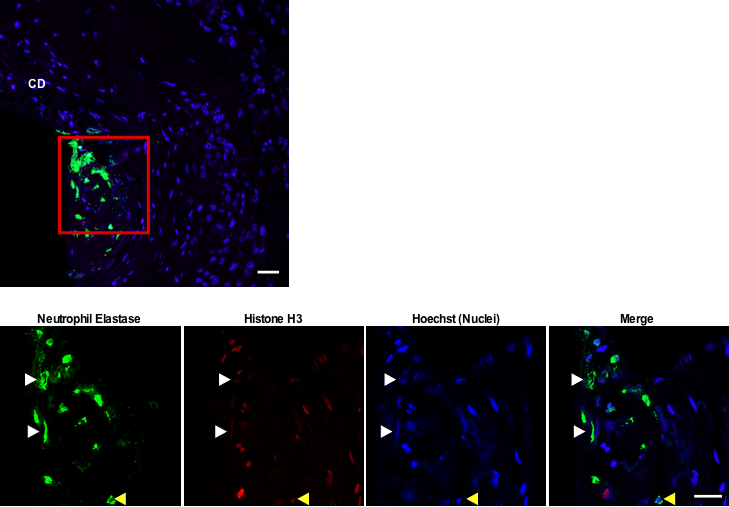

Supplement: FIG S6 [file mbo006184186sf6.tif]
